# Integrins and their potential roles in mammalian pregnancy

**DOI:** 10.1186/s40104-023-00918-0

**Published:** 2023-09-08

**Authors:** Gregory A. Johnson, Robert C. Burghardt, Fuller W. Bazer, Heewon Seo, Joe W. Cain

**Affiliations:** 1 Department of Veterinary Integrative Biosciences, School of Veterinary Medicine and Biomedical Sciences, Texas A&M University, College Station, TX 77843-4459 USA; 2 Department of Animal Science, College of Agriculture and Life Sciences, Texas A&M University, College Station, TX 77843-2471 USA

**Keywords:** Humans, Implantation, Integrins, Pigs, Pregnancy, Rodents, Sheep

## Abstract

Integrins are a highly complex family of receptors that, when expressed on the surface of cells, can mediate reciprocal cell-to-cell and cell-to-extracellular matrix (ECM) interactions leading to assembly of integrin adhesion complexes (IACs) that initiate many signaling functions both at the membrane and deeper within the cytoplasm to coordinate processes including cell adhesion, migration, proliferation, survival, differentiation, and metabolism. All metazoan organisms possess integrins, and it is generally agreed that integrins were associated with the evolution of multicellularity, being essential for the association of cells with their neighbors and surroundings, during embryonic development and many aspects of cellular and molecular biology. Integrins have important roles in many aspects of embryonic development, normal physiology, and disease processes with a multitude of functions discovered and elucidated for integrins that directly influence many areas of biology and medicine, including mammalian pregnancy, in particular implantation of the blastocyst to the uterine wall, subsequent placentation and conceptus (embryo/fetus and associated placental membranes) development. This review provides a succinct overview of integrin structure, ligand binding, and signaling followed with a concise overview of embryonic development, implantation, and early placentation in pigs, sheep, humans, and mice as an example for rodents. A brief timeline of the initial localization of integrin subunits to the uterine luminal epithelium (LE) and conceptus trophoblast is then presented, followed by sequential summaries of integrin expression and function during gestation in pigs, sheep, humans, and rodents. As appropriate for this journal, summaries of integrin expression and function during gestation in pigs and sheep are in depth, whereas summaries for humans and rodents are brief. Because similar models to those illustrated in Fig. 1, 2, 3, 4, 5 and 6 are present throughout the scientific literature, the illustrations in this manuscript are drafted as Viking imagery for entertainment purposes.

## Introduction

Integrins are receptors on the surface of cells that mediate reciprocal cell-to-cell and cell-to-extracellular matrix (ECM) interactions [1]. All metazoan organisms possess integrins, and it is generally agreed that integrins were associated with the evolution of multicellularity, being essential for the association of cells with their neighbors and surroundings, during embryonic development and many aspects of cellular and molecular biology. Integrins have not been detected in prokaryotes, plants, or fungi [2], but even the simplest invertebrates have integrins [3, 4]. The most primitive of animals that demonstrate bilateral left and right symmetry, a head and a tail, and a ventral-dorsal axis have at least two integrin alpha/beta (αβ) heterodimers and αβ-heterodimers persist in flies, nematodes, and vertebrates, with extensive expansion of the αβ-heterodimer repertoire in vertebrates [5]. Indeed, all human cells, except mature red blood cells, have one or more integrins [6]. The integrin receptor family was first recognized in the 1980s and has since become amongst the better-understood of cell adhesion receptors [7]. Integrin biochemistry is complicated. Integrins assemble into multiple αβ-heterodimer receptors, bind a large and varied array of ligands, and in addition to their role in physical adhesion they activate signaling pathways at least as complex as those activated by the tyrosine kinase and G-protein-coupled receptors, which integrins often cooperate with during cell signaling. Further, their physical connections to both the cytoskeleton on the inside of the cell and ECM on the outside of the cell mediate mechanotransduction as well as biochemical signaling [8]. Integrins have important roles in many aspects of embryonic development, normal physiology, and disease processes with a multitude of functions discovered and elucidated for integrins that directly influence many areas of biology and medicine [6]. One of these areas is mammalian pregnancy [9–11].

## Integrin structure, ligand binding, and signaling

**Fig. 1:**
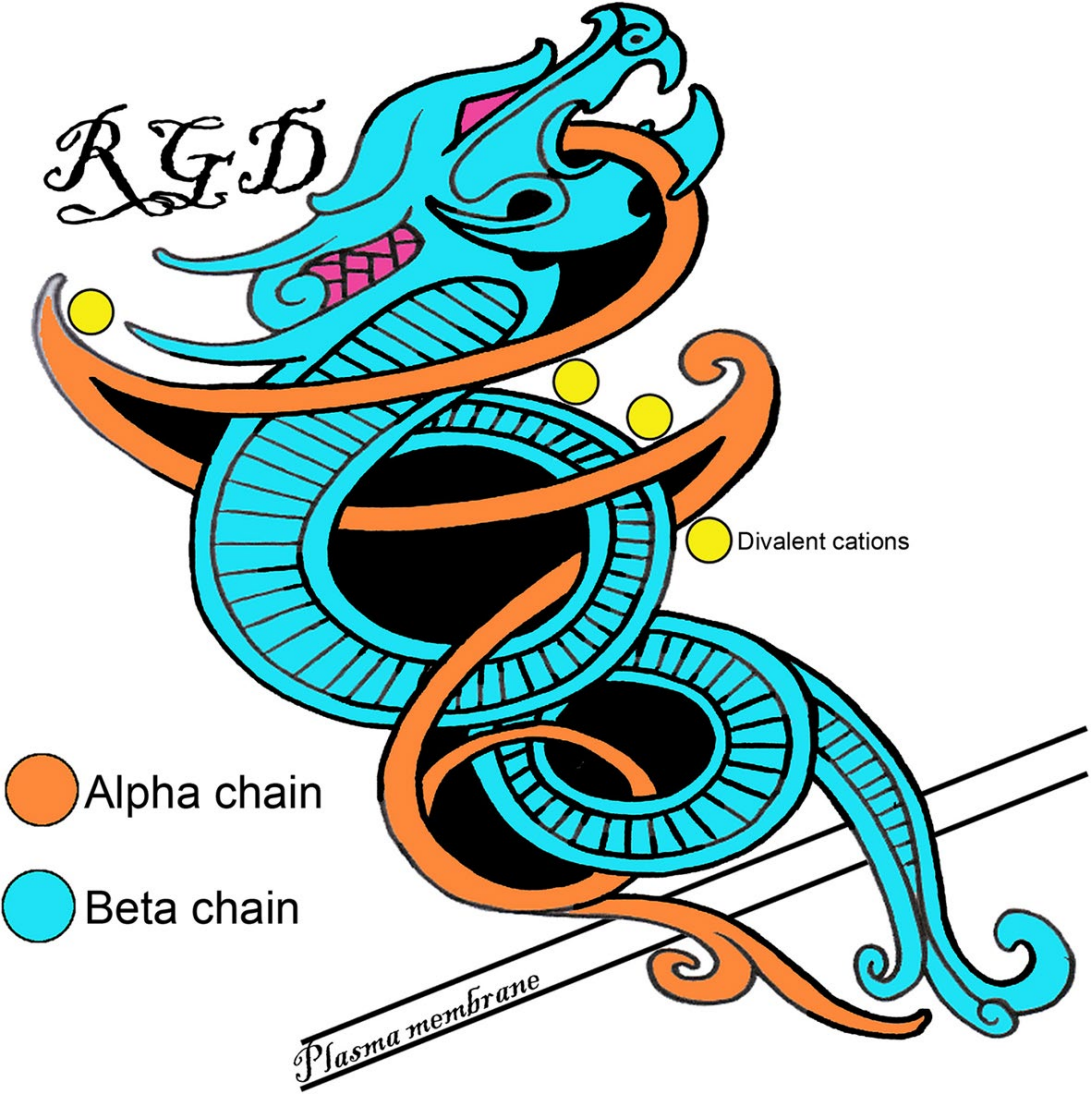
The Saga of integrins: Basic integrin structure. Integrins are dominant glycoproteins in adhesion cascades. They comprise a ubiquitous family of cation-dependent, heterodimeric [one α-subunit (chain) non-covalently linked to one β-subunit (chain)], intrinsic transmembrane glycoprotein receptors that mediate cellular differentiation, motility, and adhesion. Integrins are grouped according to the ligands they bind. Those that carry out ligand binding through integrin receptor recognition of small peptide sequences include integrins that bind arginine-glycine-aspartic acid (RGD; depicted here), leucine-aspartic-acid-valine (LDV), and glycine-phenylalanine-hydroxyproline-glycine-glutamic acid-arginine (GFOGER) within collagen. Integrins are also grouped into those that bind laminin, and leukocyte-specific receptors

**Fig. 2:**
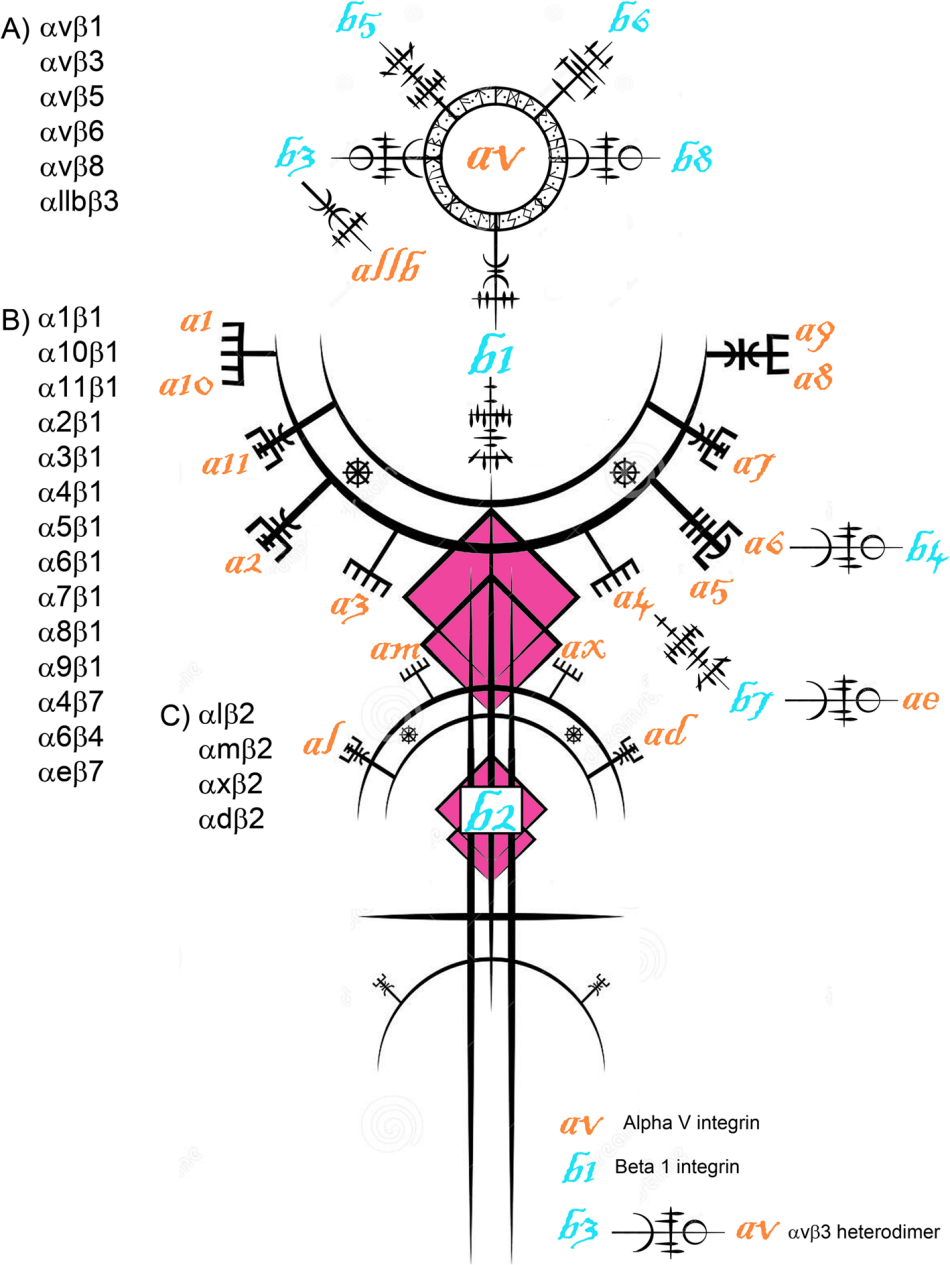
The Saga of integrins: The integrin receptor (integrin) family. The integrin family can form at least 24 distinct pairings of its 18 α-subunits and 8 β-subunits. Integrins that bind to arginine-glycine-aspartic acid (RGD) sequences in ligands include αvβ1, αvβ3, αvβ5, αvβ6, αvβ8, α5β1, α8β1, and αIIbβ3. Those that bind to laminin include α3β1, α6β1, α6β4 and α7β1. Integrins that bind the glycine-phenylalanine-hydroxyproline-glycine-glutamic acid-arginine (GFOGER) sequence in collagen include α1β1, α2β1, α10β1 and α11β1. Those that are leukocyte-specific receptors include α4β1, αLβ2, αMβ2, αDβ2 and αXβ2

**Fig. 3:**
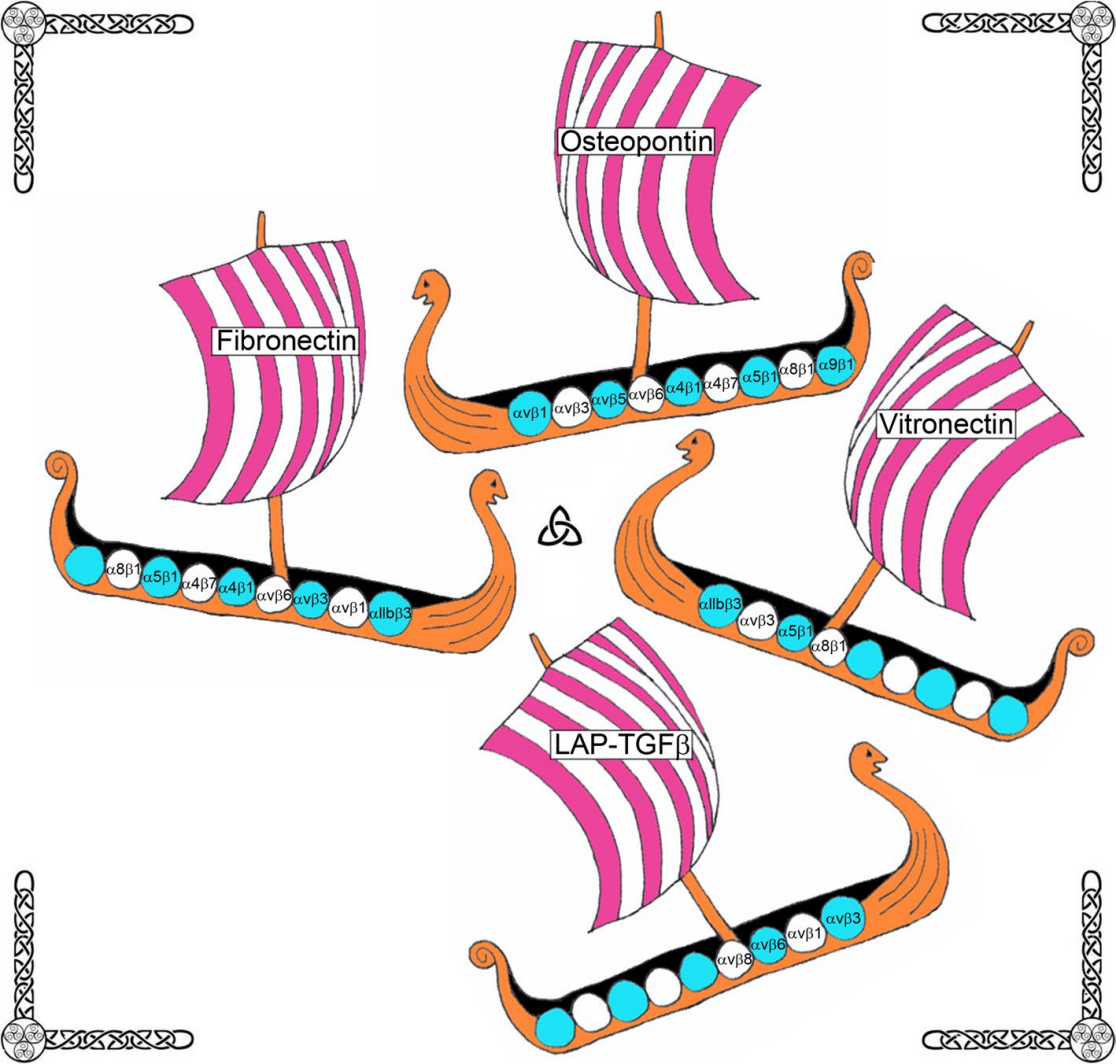
The Saga of integrins: Integrin iigands. More than one integrin receptor (integrin) can recognize a specific ECM ligand, and more than one ligand can bind a specific integrin. Depicted are Viking ships representing ECM proteins that have been localized to implantation sites in mammals carrying shields representing integrins that potentially bind to these proteins

In general, integrins consist of noncovalently linked α- and β-subunits. Each polypeptide subunit passes through the plasma membrane once, and has a large extracellular domain of greater than 1,600 amino acids, and a short cytoplasmic domain of about 20–50 amino acids [7, 12, 13]. Together these paired subunits are termed integrins (this term will be used for the remainder of this review to describe an αβ-heterodimer) or αβ-heterodimers or integrin receptors (Fig. 1). In mammals, 8 β-subunits can associate with 18 α-subunits to form 24 distinct integrins [6] (Fig. 2). Most integrins can bind to a wide variety of ligands, and many ligands can bind to multiple integrins [14] (Fig. 3).

**Fig. 4:**
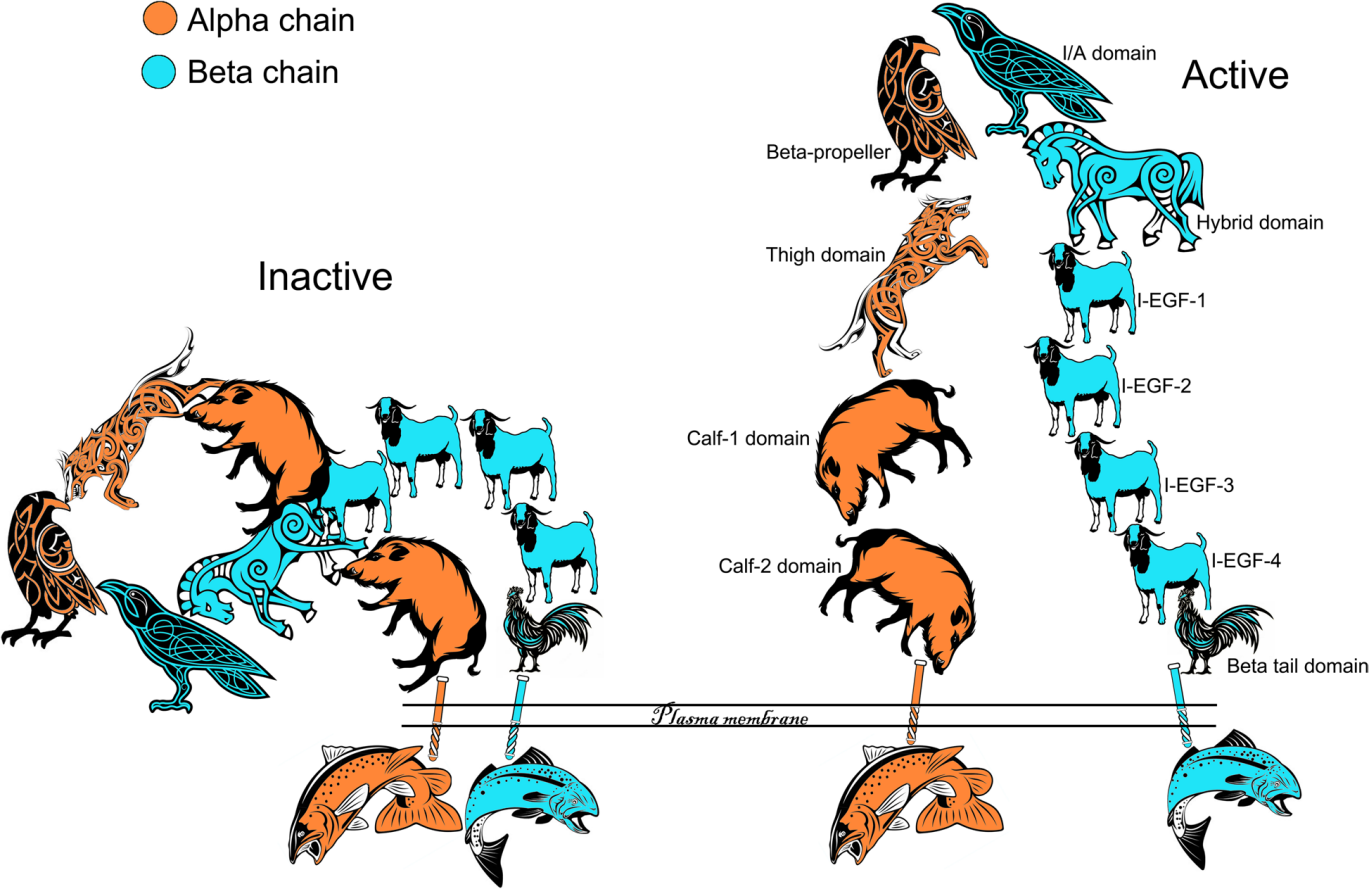
The Saga of integrins: Integrin binding and activation. Shown is the structure of the unliganded (Inactive) and liganded (Active) αvβ3 integrin with the αv-subunit (chain) in orange and the β3-subunit (chain) in blue. In the inactive form the propeller, I/A, hybrid and thigh domains are bent over towards the carboxyl terminal of the legs of the α- and β-subunits which are inserted through the plasma membrane and connected to short cytoplasmic domains. The organization of the domains are difficult to discern in this configuration but are more easily resolved in the active configuration. In the active form the α- and β-subunits are unfolded freeing the propeller and I/A domains for ligand binding. Activation of the integrin is driven by either ligand binding or by effects on the cytoplasmic domains leading to straightening of the α- and β-subunits and separation of the legs. The straightening of the legs separates the cytoplasmic domains and allows binding of cytoplasmic proteins and intracellular signaling. These changes in integrin configuration are reversible and operate in either direction, outside-in or inside-out

Using the αvβ3 integrin as an example (Fig. 4), the terminal or “head” regions of the αv and β3 extracellular domains include an α-subunit β-propeller and a β-subunit I/A domain. These domains complex together to form the ligand binding region of the integrin, and the structure of the I/A domain is that of a Rossmann fold with a core of parallel β sheets surrounded by amphipathic α helices, a structure characteristic of a large group of proteins involved in protein–protein interactions [6, 15–17]. Residues from the loops of the I/A domain coordinate a metal ion-dependent adhesion site (MIDAS) with divalent cations, and divalent cations are required for integrin binding to ligands [15]. The amino terminal of the β-propellor of the α-subunit is attached to three β-sandwich domains, the thigh, calf 1, and calf 2 of the “leg” of the subunit. Although the β-subunit I/A domain lies at the distal end of the molecule, it does not constitute the amino-terminal, rather it inserts into a loop of a β-sandwich hybrid domain. The remainder of the “leg” of the β-subunit is composed of four tandem cystine-rich repeats, the first two of which remain poorly resolved while the third and fourth are epidermal growth factor (EGF)-like folds, ending with a carboxyl terminal β-sheet β-tail domain (Fig. 4) [6, 18].

**Fig. 5:**
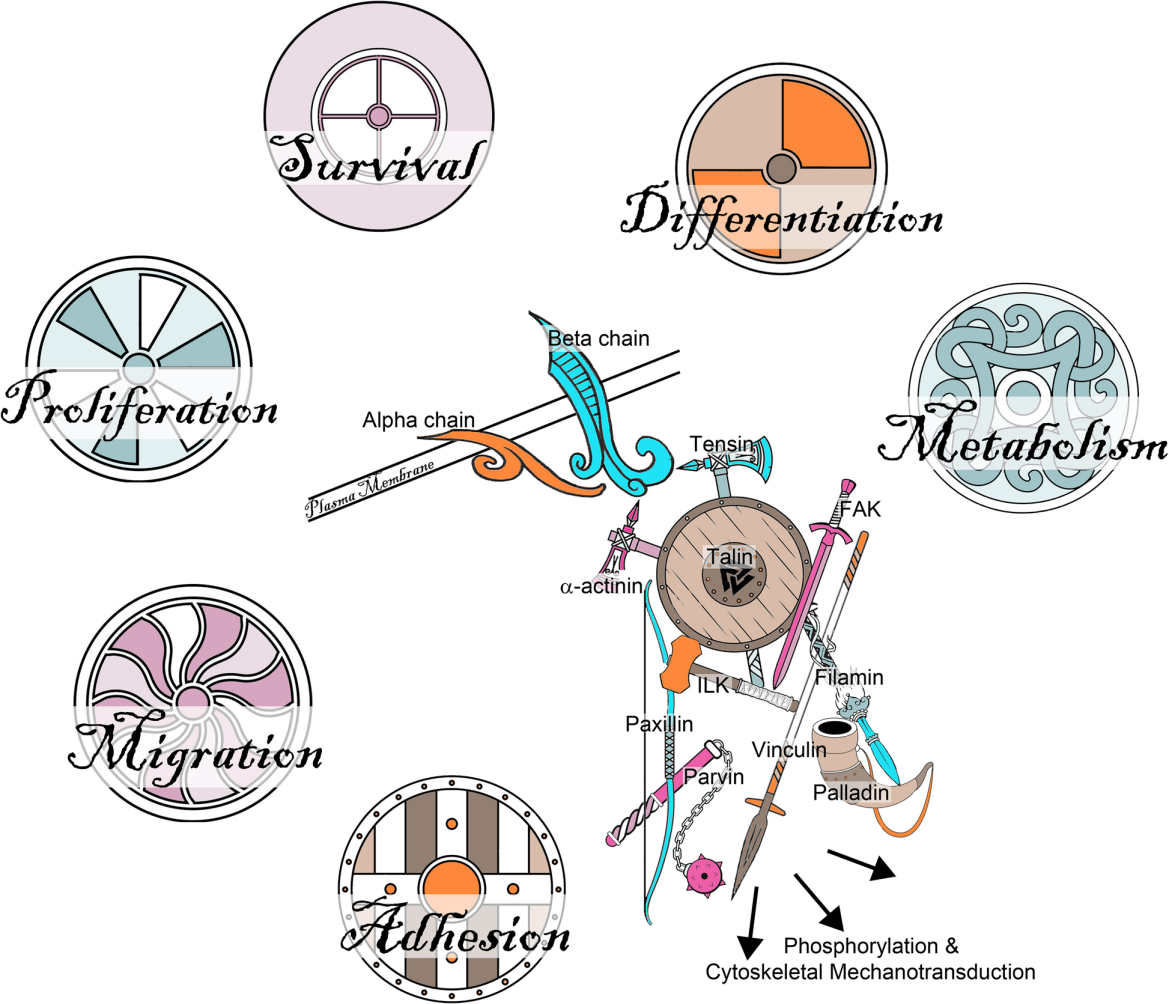
The Saga of integrins: Integrin adhesion complexes. When the extracellular domains of integrins bind to ECM ligands they cluster within the plasma membrane and the cytoplasmic domains of the integrins become closely associated with the cytoskeleton resulting in the assembly of aggregates of 1) integrins at the surface of the cell, 2) cytoskeletal proteins within the cell, and 3) proteins within the ECM which are large enough to be observed by immunofluorescence microscopy and are known as integrin adhesion complexes (IACs). The proteins recruited to IACs, the integrin adhesome, perform many signaling functions both at the membrane and deeper within the cytoplasm to coordinate processes including cell adhesion, migration, proliferation, survival, differentiation, and metabolism. The integrin adhesome is highly complex and only cryptically alluded to in the figure. The “consensus adhesome” represents links between integrins and the actin cytoskeleton and the adaptor proteins that directly link integrins with actin are α-actinin, filamin, talin, and tensin. Despite the complexity of IACs, they are dynamic, and turnover can occur within a few minutes

An important aspect of integrins is their ability to transmit signals both “outside in” and inside out” that are transmitted through large conformational changes in the extracellular domains in the integrin [6, 19–21]. Although initially thought otherwise [18, 19], it is now accepted that the subunits of inactive integrins are bent over between the thigh and calf domains of the α-subunit and in the third and fourth EGF-like regions of the β-subunit to produce a 135-degree downward angle [22, 23]. Active integrins have an extended shape and there is separation of the legs of the α- and β-subunits (Fig. 4). This strongly suggests that activation of the ligand binding domain and ligand binding are coupled to the straightening and separation of the legs of the α- and β-subunits resulting in separation of the transmembrane and cytoplasmic domains of the integrins to allow interactions with cytoskeletal and signal transduction molecules for outside-in signaling [24]. Inside-out signaling is likely mediated via the physical separation of the cytoplasmic domains of the subunits via intracellular molecules such as talin (TLN1), and perhaps others, resulting in activation of the extracellular head of the integrin for ligand binding (Fig. 5) [6]. Indeed, the head domain of TLN1 binds to the cytoplasmic domain of the β3-subunit, but not to the cytoplasmic domains of α-subunits, and in doing so separates the β3-subunit cytoplasmic tail from the α-subunit cytoplasmic tail and activates the integrin [25, 26]. Multiple proteins bind to integrin tails, and another intracellular candidate for integrin activation is focal adhesion kinase (FAK) [6].

The cytoplasmic domains of integrins lack enzymatic activity and transduce signals through cytoplasmic adaptor proteins that connect the integrin to the cytoskeleton, kinases, and growth factor receptors. As integrins bind ECM proteins they cluster within the plasma membrane and interact with many actin-associated proteins including TLN1, α-actinin, vinculin (VCL), tensin, and paxillin to organize actin filaments into larger stress fibers that result in more integrin clustering and eventual assembly of ECM proteins, integrins and cytoplasmic cytoskeletal and signaling molecules on both sides of the plasma membrane. These are termed integrin adhesion complexes (IACs) (Fig. 5) [27]. Integrins also activate various kinases, some of which phosphorylate cytoskeletal proteins to regulate cell shape and migration, and integrins recruit signaling molecules that regulate cell adhesiveness to the ECM [28]. The proteins found in IACs are called the integrin adhesome, and IACs convert spatiotemporal properties of the ECM into intra-cellular and inter-cellular/ECM signaling that has wide-ranging outcomes. The composition of IACs is complex with the potential for interactions with up to 2,412 proteins [8, 29, 30]. The “consensus adhesome” represents links between integrins and the actin cytoskeleton and includes 60 proteins that are enriched at least two-fold when binding to fibronectin (FN1) in multiple IAC datasets [31]. Some members of this “consensus adhesome” are kindlin-integrin-linked kinase (ILK)- particularly interesting new cysteine-histidine-rich protein (PINCH), FAK-paxillin, TLN1-VCL, and α-actinin-zyxin-vasodilator-stimulated phosphoprotein (VASP) (Fig. 5) [8, 30, 31].

The ability of cells to simultaneously utilize multiple integrins to bind sites exposed at differing angles within ECM networks provides the cell with a topological map of its surroundings that can be converted to intracellular signaling that supports responses to the microenvironment in the form of migration, differentiation, proliferation and/or growth, the synthesis of proteins, or altered metabolism. Therefore, the phenotype of cells can be influenced by changes in the cell’s microenvironment in powerful and eloquent ways [8]. Pregnancy, particularly implantation of the blastocyst to/into the uterine wall and early placental development, involves unique and rapid alterations to the ECM and interactions between the ECM and cells to support significant tissue remodeling required for growth and development of the conceptus (embryo and associated placental membranes). Serious consideration has been given to the involvement of integrins in these complex events. The remainder of this review focuses on the potential roles of integrins in mammalian pregnancy.

## Embryonic development, implantation and early placentation

Considerable variability exists among species relative to the histogenesis and organization of the placenta; however, placental trophoblast interactions with maternal uterine tissue remain extensive in all species, and this allows for the close juxtaposition of the microcirculatory systems of the uterus and placenta for the transport of nutrients from the mother to the embryo/fetus [32]. Although the timing and location of key events varies, the early stages of embryonic development are similar among pigs, sheep, humans, and mice/rats, the species of focus in this review. After fertilization the zygote undergoes the first cleavage division to form the 2-cell embryo, and cleavage divisions continue through the 8–16 cell stage, when transcriptome activation occurs. These divisions culminate in formation of the solid mass of cells (blastomeres), called the morula, that remains encased in the zona pellucida of the oocyte. The pluripotent blastomeres differentiate into the blastocyst consisting of an inner cell mass (ICM) and a hollow circular space, called the blastocoel, surrounded by a single layer of trophoblast cells. The ICM develops into the primitive ectoderm, mesoderm and endoderm of the embryo, and the trophoblast represents the initial placenta. Together the ICM and trophoblast are termed the blastocyst [33]. The blastocyst then hatches from the zona pellucida and the trophoblast cells of this circular blastocyst attach to the surface of the uterine luminal epithelium (LE) to begin implantation in humans and mice. In pigs and sheep, the conceptus increases in size before undergoing elongation, a rapid morphological transition from circular to tubular to filamentous forms of what is now termed the conceptus. Conceptus elongation substantially increases the surface area of placental trophoblast that attaches to the uterine LE, presumably to maximize the opportunity for nutrient and gas exchange between the conceptus and uterus in these species in which the conceptus does not invade into the uterine wall during implantation [32, 33].

**Fig. 6:**
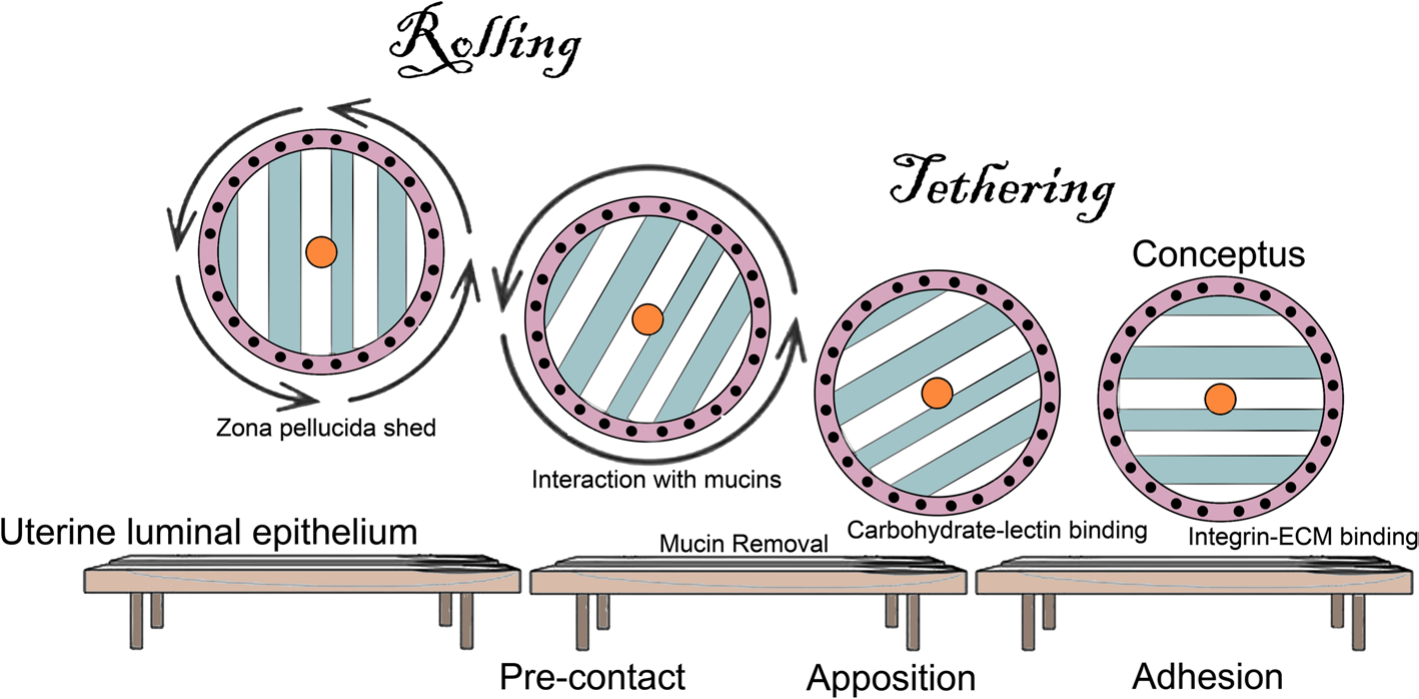
The Saga of integrins: The implantation cascade. Depicted is a generalized summary of the attachment cascade for implantation in humans in which the conceptus is hypothesized to roll across the uterine surface until it is slowed and tethered to the uterine luminal epithelium (LE) first via interactions with mucins, followed by carbohydrate-lectin binding, and completed when firm adhesion is mediated through integrins binding to ECM bridging ligands, in a manner similar to the extravasation of leukocytes from the vasculature [39, 40]. Similar cascades are postulated for pigs, sheep and mice; however, the conceptuses of pigs and sheep likely do not roll across the uterine surface. Instead, the trophoblast cells proliferate and migrate across the uterine surface as the conceptuses undergo elongation [41, 42]

Perhaps nowhere else in the mammalian body do the apical domains of the surface epithelium of one organ physically attach to the apical domains of the surface epithelium of another organ. This arrangement is unique to pregnancy. Thus, it is not surprising that attachment of the conceptus to the uterine LE to initiate implantation is highly synchronized and requires reciprocal secretory and physical interactions between a developmentally competent conceptus and the uterus during a restricted period of the uterine cycle termed the “window of receptivity” [34]. Interactions between the apical surfaces of the uterine LE and trophoblast progress from a non-adhesive or pre-contact phase to an apposition phase and conclude with adhesion. Conceptus attachment first requires the removal of mucins from the glycocalyx of the uterine LE that sterically inhibit adhesion. The removal of these mucins allows for direct physical interactions between a mosaic of carbohydrates and lectins at the apical surfaces of the opposing uterine LE and conceptus trophoblast cells which contribute to initial attachment of the trophoblast to the uterine LE [35, 36]. These low affinity contacts are then strengthened by a repertoire of adhesive interactions between integrins and ECM molecules that appear to be the dominant contributors to stable adhesion for implantation [37–39] (Fig. 6 [39–42]).

Implantation can be defined as the beginning of placentation, and placentation differs significantly among species [43, 44]. Pigs have a true epitheliochorial placenta in which separation of placental and uterine vasculatures is always maintained representing a substantial barrier to hemotrophic nutrient transport from the mother to fetus. To overcome this, the interhaemal distance is minimized through degradation of much of the connective tissue separating the uterine LE and trophoblast from their underlying capillary beds, the uterine-placental interface is extensively remodeled to form folds that increase the area of uterine-placental association across the entire placenta, and pockets of the trophoblast lining of the placenta that are not attached to the uterine LE, called areolae, form at the openings of the uterine glands to receive histotroph [42, 45, 46]. Sheep have synepitheliochorial placentae in which trophoblast cells migrate into the uterine LE and fuse with other cells to form multinucleated syncytia. The placenta organizes to form placentomes in which highly branched villous placental folds protrude into crypts in the uterine caruncular tissue, and interplacentomal regions are characterized by uterine glands and areolae [41, 47]. Humans have hemochorial placentae in which restricted displacement of uterine LE cells allows syncytiotrophoblast cells to invade into the uterine stroma and leave open lacunae in their wake, followed by a second wave of invasion by cytotrophoblast cells that remodel uterine spiral arteries to facilitate the flow of blood into the lacuna. The uterine stroma decidualizes in response to maternal production of progesterone [40, 48, 49]. Mice also have a hemochorial placenta in which signals from the trophoblast that attaches to the uterine LE initiate decidualisation of the uterine stroma. Then the uterine LE at sites of implantation undergo cell death or entosis as trophoblast cells invade into the uterus with primary trophoblast giant cells aligning adjacent to the decidua, and eventual fusing of spongiotrophoblast with the allantois forms a labyrinthine layer in which extensive intermingling of maternal blood and placental blood vessels occurs [49, 50]. Clearly conceptus implantation and placental development requires extensive cell adhesion, proliferation, migration and morphogenesis, all activities potentially mediated through the actions of integrins.

## A brief timeline of the initial localization of integrin subunits to the uterine LE and conceptus trophoblast

It is generally accepted that integrins expressed at the apical surfaces of conceptus trophoblast and uterine LE cells bind bridging ligands to attach the conceptus to the uterus for implantation, but in the early 1990s this was a unique and somewhat unprecedented role to propose for integrins because the apical surface of epithelial cells is not normally a site of intercellular interactions. In 1992, two studies localized multiple integrin subunits within the endometrium of women during the menstrual cycle, and one study noted that expression of some of these integrins was regulated by stage of the menstrual cycle. The integrin subunits α1, αv, and β3 increased in endometria during the secretory phase of the menstrual cycle and β3 was localized to uterine LE; however, an apical distribution of the integrin subunits was not demonstrated [51]. The second study reported localization of the α1-, α3-, α5-, α6- and β1-subunits to the uterine LE of secretory stage endometria, but, again, apical distribution of these subunits was not confirmed [52]. In the same year the first integrin subunit, β1, was found to be expressed by human blastocysts prior to implantation with particularly intense immunostaining observed in hatching blastocysts [53]. In 1993, the localization of the α3- and α6-subunits to the uterine LE of women was again reported, and the α2- and α4-subunits were added to the list of integrin subunits expressed by uterine LE [54]. Also, in 1993, Sutherland and co-workers examined integrin expression by the trophoblast of mouse blastocysts during development through the peri-implantation period of pregnancy. Although multiple integrin subunits were shown to be expressed by blastocysts, only αvβ3 integrin was confirmed to be present at the apical surface of trophoblast cells by immunofluorescence microscopy [55]. Significantly, in 1994, Aplin and co-workers clearly demonstrated the presence of the β5-subunit at the apical domain of the uterine LE of women where β5 could potentially serve at a mediator of attachment of uterine LE to trophoblast [56]. By 1995 this same research group localized αv-, α3-, β1-, β3-, and β5-subunits on human blastocysts [57]. Bowen et al. [58] established the presence of multiple integrin subunits at the apical surfaces of the uterine LE and conceptus trophoblast of pigs. The integrin subunits α4, α5, αv, β1, β3 and β5 were localized to porcine implantation sites on d 12 through 15 of gestation [58]. In 1999, the integrin subunits αv and β3 were localized to the apical surfaces of the uterine LE and conceptus trophoblast on d 16 of gestation, during the attachment phase of implantation in sheep, and in 2001 αv-, α4-, α5-, β1-, β3- and β5-subunits were shown to be constitutively expressed on these tissue surfaces during the peri-implantation period of pregnancy [59, 60].

## Summary of integrin expression and function during gestation in pigs

Progesterone dominates the uterine environment during the establishment of pregnancy in pigs, but other factors are required to maintain a successful pregnancy. These include the secretion of estrogens, prostaglandins, interleukin-1 beta and interferons gamma and delta from the conceptus, and secretions of histotroph from the uterine LE and glandular epithelium (GE) (reviewed in [32, 42, 61–69]. Within this complex environment porcine conceptuses undergo the most extensive elongation of any species that has been studied in depth. The day 10 blastocyst develops a dense band of cells called the elongation zone, composed of both trophoblast and endoderm, that extends from the ICM to the tip of the ovoid blastocyst. Elongation of the trophoblast and endoderm then begins and within the span of a few hours the rate of elongation increases to 30 to 45 mm/h from the 10 mm blastocyst to the 100 to 200 mm long filamentous conceptus. There is further rapid elongation to 800 to 1,000 mm in length by d 16 of pregnancy mediated through alterations in the microfilaments and junctional complexes of trophoblast cells and formation of filopodia by endodermal cells. This last period of elongation involves both cell proliferation and cellular hyperplasia, and each conceptus within the litter achieves maximum surface area for contact between trophoblast and uterine LE to facilitate uptake of nutrients from uterine LE and uterine GE, which increase coincidentally with elongation of the conceptuses [62]. Overlapping these elongation events is the attachment phase of implantation which roughly occurs from d 13 to 26 of pregnancy in pigs. Throughout implantation, the glycocalyx that extends from the apical surface of the uterine LE is thicker than at the surface of the trophoblast. On d 13 and 14, the uterine LE develops protrusions that become enclosed by caps of trophoblast cells that serve to physically immobilize the conceptus, and by d 14 there is close apposition between the apical plasma membranes of trophoblast and uterine LE. Interdigitating microvilli form between these plasma membranes on d 15 and 16, and then the interface becomes increasingly complex between d 15 and 20 of pregnancy. This transition is characterized by the development of apical domes on uterine LE closely apposed to the trophoblast and long cytoplasmic extensions into a luminal space between the apical domes. Finally, adhesion transitions into placentation through ever-increasing development of interdigitating microvilli occurs between the uterine LE and trophoblast by d 26 of gestation [70].

Although it has been demonstrated that progesterone downregulates expression of mucin-1 (MUC1) at the apical surfaces of uterine LE [58, 71], interactions between carbohydrates and lectins during the adhesion cascade of pigs have not been systematically investigated. However, it is likely that these carbohydrate ligands and their lectin receptors, expressed at the apical surfaces of the conceptus trophoblast and uterine LE, undergo a series of attach-and-release events resulting in maximal apposition of the conceptus trophoblast to the uterine LE, similar to “rolling and tethering” proposed for the initial attachment of human blastocysts to the uterine wall [40]. These low affinity contacts are then likely stabilized by adhesion between a repertoire of integrins and ECM proteins [37, 72]. Eight integrin subunits are expressed at the apical surface of both the conceptus trophoblast and the uterine LE of pigs. These include α1, α3, α4, α5, αv, β1, β3 and β5. The expression of the α4-, α5- and β1-subunits on the uterine LE increases during the peri-implantation period of pregnancy, and treatment with progesterone increases the expression of these integrin subunits at the apical surface of uterine LE cells of cyclic pigs [58]. In vitro studies strongly support these in vivo data. Trophoblastic vesicles generated from d 12 and 15 porcine conceptuses and polarized uterine LE cells grown on matrigel express all of the integrin subunits observed on conceptuses. Treatment of these cultures with estrogen results in the upregulation of α1-, α4- and α5-subunit expression, treatment with progesterone results in upregulation of the α1-, α4-, α5- and β1-subunits, and co-treatment with estrogen and progesterone upregulates the α1-, α3-, α4-, α5- and β1-subunits [71]. It is noteworthy that immunofluorescence microscopy has localized the integrin subunits α4, α5, αv, β1, β3 and β5 at sites of implantation on d 12 through 15 of gestation [58]. Further, immunofluorescence microscopy strongly suggests that the αv-, β1-, β3- and β5-subunits incorporate into IACs at sites of implantation in pigs, because the staining pattern of each of these subunits revealed large aggregates at the apical domains of uterine LE and trophoblast from sites of implantation on d 16 through 24 of gestation [73, 74].

Collectively, the subunits detected in the uterine LE and trophoblast of pigs have the potential to assemble into the αvβ1, αvβ3, αvβ5, α4β1 and α5β1 integrins, and these integrins may function in adhesion with FN1, vitronectin (VTN), the latency associated peptide (LAP) of transforming growth factor beta (TGFβ), the inter-α-trypsin inhibitor heavy chain-like protein (IαIH4), and/or osteopontin [OPN, secreted phosphoprotein 1 (SPP1)] expressed at the uterine-placental interface of pigs [37]. Three integrins expressed by porcine uterine LE and trophoblast, αvβ3, α4β1 and α5β1 (see Fig. 3) can bind FN1, and the αvβ3 integrin is considered the major VTN receptor. Both FN1 and VTN are present in uterine flushings during the peri-implantation period and at sites of implantation in pigs [58]. TGFβ is released from cells in a latent form due to non-covalent association with the LAP. This LAP contains an RGD sequence that can bind to the αvβ1 and αvβ3 integrins present at implantation sites, and TGFβ activity, and therefore the availability of LAP to bind integrins on uterine LE and conceptus trophoblast, increases within the intrauterine environment during the peri-implantation period of pigs [75]. The IαIH4 protein contains a von Willebrand type A domain that is a recognition site for the αvβ3 integrin receptor, and both IαIH4 protein expression and kallikrein enzymatic activity, increase within the uterine environment during the peri-implantation period of pregnancy in pigs [76, 77]. OPN is the most promiscuous of these ligands and interacts with αvβ1, αvβ3, αvβ5, α4β1 and α5β1 integrins. It has been established that OPN is prominent within the intrauterine environment of pigs during the peri-implantation period of pregnancy [77–80].

Affinity chromatography followed by immunoprecipitation was used to demonstrate the direct binding of specific integrins to ligands on porcine uterine epithelial (pUE) and trophoblast (pTr2) cells [73, 74]. Detergent extracts of surface-biotinylated pUE and/or pTr2 cells were incubated with either LAP-Sepharose or OPN-Sepharose and the proteins that bound to LAP and OPN were eluted with ethylenediaminetetraacetic acid (EDTA) to chelate cations and release bound integrins. For the LAP experiments, the eluted fractions from pTr2 extracts were subjected to immunoprecipitation using antibodies to the integrin subunits αv, β1, β3, β5, β6, and β8, and each of these subunits was shown to bind LAP [74]. For the OPN experiments, the eluted fractions from both pUE and pTr2 extracts were subjected to immunoprecipitation using antibodies to the integrin subunits αv, α4, α5, β1, β3, β5 and β6, and it was determined that the αvβ6 integrin on pTr2 cells and αvβ3 integrin on pUE cells directly bound to OPN [73]. Integrin binding to OPN promoted dose-, RGD-, and cation-dependent attachment of pTr2 and pUE cells, and stimulated haptotactic pTr2 cell migration directionally along a physical gradient of nonsoluble OPN [73]. Knockdown of the αv-subunit in pTr2 cells by siRNA reduced pTr2 attachment to OPN and FN1, but did not affect attachment to type I collagen, as this protein does not bind to αv-subunit-containing integrins (see Fig. 3) [81]. Finally, the αv-subunit co-localized with TLN1 in IACs generated at the apical domain of pTr2 cells around OPN-coated microspheres briefly cultured at the top of the cells [60, 73]. Collectively, results support that integrins directly bind OPN on pUE cells, and bind to LAP, FN1 and OPN on pTr2 cells, and this binding stimulates attachment to FN1 and OPN, and OPN-mediated haptotactic cell migration, and IAC assembly.

Integrin expression at the uterine-placental interface of pigs is not limited to the peri-implantation period of pregnancy. Integrin mRNAs for the subunits αv, α2, β1, β3, β5, β6, and β8 have been detected in both endometrial and placental tissues from Days 30–90 of gestation in pigs [82]; however, although αv-, β3- and β6-subunit mRNAs and proteins are present at the uterine-placental interface through d 60 of gestation, IACs containing these subunits are not observed by day 50, suggesting that as placentation progresses, subsequent folding at the uterine-placental interface disperses the mechanical torsion forces that drive IAC assembly [81]. Therefore, it is possible that the roles of integrins go beyond physical attachment and cell migration, and integrins may utilize other signaling cascades within the integrin adhesome to influence the uterine-placental environment. Two recent studies established that integrins mediate the ability of OPN to potentially affect angiogenesis and ion transport in uterine and placental tissues of pigs [83, 84]. Angiogenesis is fundamental to the expansion of the placental vasculature during pregnancy, and both integrins and OPN are associated with vascular development [85–89]. Angiogenic blood vessels emerge from differentiated adult endothelial cells that line the pre-existing vasculature; however, circulating endothelial progenitor cells (EPCs) released from bone marrow can migrate and incorporate into newly vascularized tissue where they differentiate into mature endothelium [90, 91]. When porcine EPCs were cultured on OPN-coated slides and placed into collagen gel invasion assays [92] supplemented with OPN, the αv-subunit was observed in IACs at the basal surface of the EPCs that enhanced their incorporation into human umbilical vein endothelial cell (HUVEC) sprouts. Silencing of the αv-subunit in EPCs using siRNA reduced EPC binding to OPN, IAC assembly, and EPC incorporation into growing endothelial cell networks, suggesting the possibility that during placental angiogenesis EPCs expressing integrins containing the α-subunit bind to OPN through these αv-containing integrins as they incorporate into the growing vasculature to potentially augment the rate and magnitude of angiogenesis [83].

The placenta is a low permeability barrier, and effective transport of substances depends on specific transport mechanisms. Active transport requires that ions or nutrients be moved against an electrical and/or concentration gradient. In pigs, active transport of ions occurs across the chorioallantois to produce an electrochemical gradient that changes throughout gestation [93, 94]. Ussing chambers were utilized to measure transchorioallantoic voltage potential as an index of ion transport across the chorioallantoic placenta of pigs. When intact recombinant OPN was added to the mucosal side of the tissue within the Ussing chamber, an increase in the transepithelial voltage was observed; however, when recombinant OPN in which the integrin binding RGD sequence was mutated to RAD was added into the Ussing chamber, there was no increase in ion transport across the chorioallantois. This suggests that integrins expressed by the chorionic epithelium bind the RGD sequence on OPN to alter the magnitude of and/or cellular localization of nutrient transporters and/or the activity of those transporters to increase nutrient transport across the chorionic and allantoic membranes to the placental vasculature [84]. Integrins form macromolecular complexes that localize ion channels to the plasma membrane and they regulate potassium and calcium channels in multiple cell types, but ion transport across epithelia in general, and chorion in particular, had not been linked previously to integrin activation [84, 95–97].

## Summary of integrin expression and function during gestation in sheep

During the establishment of pregnancy in sheep, conceptus mononuclear trophoblast cells secrete interferon tau (IFNT) which acts on uterine LE/shallow GE (sGE) to block increases in estrogen receptor α to preclude oxytocin receptor expression. This prevents oxytocin from inducing luteolytic pulses of prostaglandin F2 alpha (PGF_2_α) and ensures maintenance of the corpus luteum (CL) for the production of progesterone, the hormone of pregnancy. The unattached sheep blastocyst is responsive to molecules supplied by the endometrium in the form of histotroph. Amongst these molecules are prostaglandins, glucose, fructose, and arginine and OPN that stimulate the mechanistic target of rapamycin (MTOR) nutrient sensing system (reviewed in [32, 41, 98–109]. Within this complex environment, the developing embryo forms the blastocyst by day 6 and the pluripotent blastomeres begin to differentiate into the ICM and trophoblast. The blastocyst hatches out of the zona pellucida between days 8 and 9 when it is about 200 µm in diameter and contains about 300 cells. It then increases in size to 400–900 µm in diameter containing about 400–900 cells, and then undergoes a rapid morphological transition called elongation [33]. The small spherical conceptus grows into a tubular form by day 11, followed by a phase of rapid growth and elongation between days 12 and 16 to form the mature filamentous conceptus of 10–22 mm on d 12, 10 cm on d 14, and 25 cm on d 17. During the early elongation period, the conceptus remains unattached to the uterine LE and is dependent on nutrients in the uterine lumen. The filamentous conceptus remains closely associated with the uterine LE until it becomes immobilized within the uterine lumen by d 14, although the conceptus can still be recovered intact from the uterus by lavage with only superficial damage. Apposition begins near the ICM, and spreads towards the ends of the elongated conceptus. By d 16 the conceptus trophoblast is firmly attached to the uterine LE with significant interdigitation between the microvilli on uterine LE and conceptus trophoblast cells, as well as between placental papillae that extend down into the lumen of the ducts of uterine glands. The conceptus attaches to both the caruncular and intercaruncular regions of the uterus, and attachment is complete by d 22 [99, 105]. The current consensus for the attachment cascade in sheep includes downregulation of MUC1 across the entire uterine surface, which “unmasks” glycosylation dependent cell adhesion molecule 1 (GlyCAM-1), galectin 15 (LGALS15) and OPN for interaction with lectins and integrins. Initial attachment is likely mediated by GLYCAM1 and LGALS15, and firm attachment is likely mediated by OPN binding integrins [11, 59, 60, 99, 106–109].

The integrin subunits αv, α4, α5, β1, β3 and β5 are constitutively present on the uterine LE and conceptus trophoblast during the peri-implantation period, and these integrin subunits potentially contribute to the assemblage of αvβ3, αvβ1, αvβ5, α4β1 and α5β1 integrins [59, 60]. Although the α5-subunit is present in the cytoplasm and only minimal protein is evident at the apical surface of uterine LE, the αv-, α4-, β1-, β3- and β5-subunits are all present at the apical surface of uterine LE during the critical period of conceptus attachment for implantation. Somewhat surprisingly, the β3-subunit is limited to the apical surface of uterine LE during the peri-implantation period, and is not expressed thereafter, suggesting that the β3 integrin may play a unique role amongst the integrins during implantation in sheep [110, 111]. When translation of mRNA for trophoblast-expressed β3 integrin was blocked through infusion of morpholino antisense oligonucleotides into the uterine lumen of pregnant ewes on day 9, sheep conceptuses elongated and implanted to the uterine wall, but embryonic growth to d 25 was inhibited, and there was decreased expression of OPN and nitric oxide synthase 3 (NOS3) in the developing allantois. This suggests effects of integrins containing the β3-subunit on development of the vasculature in the allantois required to transport nutrients from the uterus to support growth and development of the embryo [112]. Expression of the β5-subunit extends from the uterine LE into the sGE where it potentially interacts with trophoblast papillae, thought to serve as tethers against which forces necessary to generate elongation are applied, and serves as sites of maximal uptake of nutrients in histotroph [111].

It is reasonable to hypothesize that the continuous thin layer of immunostaining for αv, α4, β1, β3 and β5 at the apical surface of uterine LE from d 11 through 16 represents potential roles for the αvβ3, αvβ5 and α4β1 integrins during conceptus elongation and the adhesion cascade of implantation. Affinity chromatography and immunoprecipitation experiments determined whether αv-, α4-, α5-, β1-, β3-, β5- and β6-subunits expressed by ovine trophoblast cells (oTr1) directly bind OPN. In these experiments detergent extracts of surface-biotinylated oTr1 cells were incubated with OPN-Sepharose and the proteins that bound to OPN were eluted with EDTA to chelate cations and release bound integrins. The eluted fractions from oTr1 extracts were then subjected to immunoprecipitation using antibodies to the integrin subunits αv, α4, α5, β1, β3, and β6, and to the αvβ3 and αvβ5 integrins. Successful immunoprecipitation of labeled oTr1 integrins occurred with antibodies to the αv-, α5-, and β3-subunits, as well as an antibody to the integrin αvβ3 heterodimer. Antibody to the αv integrin subunit also precipitated a β-subunit, presumed to be β3, as an antibody to the β3 integrin subunit precipitated an α-subunit at the same relative size as the bands precipitated by the antibody to the αvβ3 heterodimer, indicating the αvβ3 integrin on oTr1 cells that binds OPN. The β1-subunit was not observed in the biotinylated oTr1 cell extracts eluted from the OPN-Sepharose column; however, the fact that the β1-subunit is the only known binding partner for α5, and α5β1 accumulates in IACs at the base of oTR1 cells cultured on OPN-coated slides suggests that the α5β1 integrin is also an active receptor for OPN in the trophoblast of sheep [113]. Further studies established that 1) RGD-mediated interaction between integrins and OPN stimulate robust oTr1 cell adhesion that was blocked when cations were removed from the culture media and in the presence of function-blocking antibodies specific for the αvβ3 integrin; 2) the αv-subunit incorporates into TLN1-containing IACs around OPN-coated microbeads at the apical surface of oTr1 cells; and 3) integrins interact via the RGD sequence of OPN to activate MAPK and p70 ribosomal protein S6 kinase beta-1 (P70S6K). Those results suggest that integrin-mediated crosstalk between the FK506-binding protein 12-rapamycin-associated protein 1 (FRAP1)/MTOR pathways and the MAPK/extracellular signal regulated kinase (ERK) pathways [113]. Finally, the accumulation of the β3-subunit and phosphorylated FAK in IACs at the base of cultured oTr1 cells increases in response to the treatment of cultured cells with the combination of arginine and soluble OPN [114].

The three tissue compartments of the uterus of sheep ie., the LE, stroma, and myometrium, exhibit tissue-specific organization of IACs during pregnancy. In the first uterine compartment, it is noteworthy that by day 40 of gestation the punctate apical surface staining of integrin-subunits identified in peri-implantation uterine LE and conceptus trophoblast [60] is replaced by large, scattered aggregates of IAC-associated αv-, α4-, β1-, and β5-subunits at the surface of the interplacentomal uterine LE and trophoblast/chorion cells [110]. The IACs are observed only in gravid uterine horns of unilaterally pregnant sheep, demonstrating a requirement for trophoblast attachment to uterine LE, and aggregates increase in number and size through d 120 of pregnancy [110]. It is noteworthy that similar IACs containing the β1-subunit are observed in the placentomes of cattle, and epithelial cells isolated from bovine placentomes bind to FN1, laminin, or type IV collagen to form IACs in culture [115]. Interestingly, in sheep, no accumulation of the β3-subunit is observed even though the αvβ3 integrin is a prominent component of the uterine-placental interface during the peri-implantation period of pregnancy in sheep [60]. In some regions of the interplacentomal uterine-placental interface, greater aggregation of integrin subunits occurs on the uterine LE, in other regions, aggregates are predominant on the placental chorion, and in some regions both uterine LE and placental chorion exhibit prominent IACs. The apparent disorganized distribution of these IACs may have a structural/physiological basis. As the uterine LE erodes during syncytialization of the of the uterine-placental interface [47], the sGE at the mouths of the uterine glands never degrades and may serve as a stable reserve of epithelial stem cells to replace the uterine LE that is lost during this period of placentation. Indeed, a similar process occurs for replacement of the surface mucous cells of the stomach by stem cells that migrate from the necks of the gastric glands, and for the replacement of enterocytes by stem cells that migrate from the crypts of Lieberkuhn in the intestines. The sGE cells proliferate, express integrins, and express OPN, but *OPN mRNA* is not detectable at the uterine-placental interface of sheep and is only present in the uterine GE [116]. It is reasonable to propose that OPN is secreted from the uterine GE and binds to the integrins expressed by the proliferating sGE. The sGE then utilize the interactions between integrins and OPN to migrate out of the uterine GE and repopulate the uterine LE in the interplacentomal regions of sheep during placentation [111]. In this capacity, the IACs interact with the actin cytoskeleton to give the chorionic cell traction as it migrates along the ECM. At the leading edge of the migrating cells, there are nascent, immature, focal complexes formed that then mature into IACs as the cells become stably attached to the ECM and more force is exerted on the focal complex [28, 117].

By day 60 of pregnancy, the interplacentomal uterine-placental interface stabilizes into a continuous seal between the uterine LE and chorion except at the openings of the uterine GE where the chorion does not fuse with the uterine LE and instead forms a pocket, referred to as an areola, to receive secretions from uterine GE. In order to maintain the integrity of these areolae there must be tight attachment between uterine LE and chorion in non-areolar regions of the interplacentomal endometrial-placental interface. In these regions of the uterine-placental interface IACs containing the αv-, α4-, α5-, β1- and β5-subunits have a well-organized pattern of expression in which the integrin subunit localizes to IACs at the apical surfaces of both uterine LE and chorionic epithelia, resulting in a gap between the apposed surfaces where adhesive ECM molecules could reside [111]. It is proposed that the temporal and spatial formation of these mature IACs represents engagement of integrins with the ECM to stabilize adhesion between uterine LE and chorionic epithelium in response to the increasing mechanical stress being placed on this interface by the ever-increasing size of the fetus and volumes of fetal fluids [110]. Interestingly, OPN co-localizes with these IACs, suggesting that OPN is acting as a bridging ligand between IACs to maintain contact between uterine LE and chorion [111].

In the second uterine compartment, during and immediately after the conceptus attaches to the uterine LE for implantation, the fibroblasts of the stratum-compactum stroma of sheep differentiate into a myofibroblast phenotype associated with upregulation of the cytoskeletal proteins desmin, vimentin, and alpha-smooth muscle actin to augment the contractility of fibroblasts [118]. These stromal cells also express the αv- and β3-subunits, as well as the ECM proteins OPN, FN1, and VTN [110]. The αvβ3 integrin that potentially assembles within the stratum compactum stroma is capable of binding VTN, FN1, and OPN to form IACs, and a diffuse spatial pattern of localization of αvβ3 integrin, VTN, FN1, and OPN within the stroma suggests they are organized into 3D matrix adhesions that developed in a mechanically stressed but a relatively strain-shielded environment [110].

In the third uterine compartment, the myometrium, the smooth muscle cells respond to forces arising during pregnancy including increases in fetal growth/weight, placental fluid volumes, and blood flow to transform the myometrium into a tissue capable of forcefully expelling the fetus and placental membranes during parturition [119]. One of the ways that smooth muscle cells respond to extracellular mechanical forces is through the assembly of integrin IACs that provide a scaffold through which cells sense and transduce responses to those forces. IACs develop at the intracellular boundary where transmembrane integrin receptors bound to ECM proteins connect with the actomyosin cytoskeleton and nucleate cytoplasmic signaling hubs [120, 121]. Indeed, in both humans and rats highly ordered IACs develop between myometrial smooth muscle cells during late pregnancy in order for the uterus to develop the mechanical strength to expel the fetus and placenta at parturition, and these IACs subsequently disassemble after parturition [122–124]. Recently it was reported that the ovine myometrium assembles IACs involving the association of the α5β1 integrin with the ECM protein FN1 and intracellularly with VCL and TLN1 during the first trimester of pregnancy [125]. These IACs increasingly organize into linear strands along the long axis of the myometrial cells as the fetus continues to grow and allantoic and amniotic fluids continue to accumulate. Mechanical stretch of the uterine wall contributes a sustained local force by day 40 of pregnancy that advances development of increasingly ordered IACs between the myometrial smooth muscle cells until parturition. This ordered structure is lost by day 1 postpartum, but the abundance of the integrin proteins remains elevated for at least two weeks postpartum [125].

## Summary of integrin expression and function during gestation in humans

The initial stages of development of the human conceptus from conception to morula occur within the oviduct, and it is the morula that enters the uterus 2 to 3 d after fertilization. The morula then quickly differentiates into the blastocyst consisting of a fluid-filled inner cavity containing the ICM and surrounded by the trophoblast. This blastocyst hatches from the zona pellucida within 72 h of entering the uterus exposing the surface of the trophoblast for interaction with the uterus both physically and in the form of uptake of histotroph. Implantation occurs about a week after ovulation [40]. The earliest stages of trophoblast adhesion to and penetration through the uterine LE have never been documented in the human [126]; however, investigators have utilized various in vitro and ex vivo systems to lead the way in our understanding of the cell adhesion cascade at implantation in which initial weak interactions between the conceptus trophoblast and uterine LE is reversible in advance of a later more stable adhesion [10, 49]. Although upregulated by progesterone during the menstrual cycle, MUC1 downregulates locally beneath the attaching blastocyst [127, 128]. This is followed by tenuous interactions between the uterine LE and trophoblast mediated by, but not limited to, mucin-associated fucosylated glycans sulpho-sialyl Lewis X, Lewis X, Lewis Y, H type 1, trophinin, heparin-binding epidermal-like growth factor (HB-EGF) and dystroglycan [49]. Stable adhesion of the conceptus to the uterine LE is then mediated through the integrins [10].

Between 1992 and 1996, Lessey and co-workers reported results from a series of studies that established integrins as important features of the human endometrium. Initial examination found that the integrin subunits αv, α1, α2, α3, α6, β3 were expressed by epithelia with the αv-subunit increasing during the secretory phase and the α1- and β3-subuits present only during the secretory phase of the menstrual cycle [9]. Further characterization of the uterine epithelial expression of integrin subunits provided evidence that the timing of maximal expression of the αv-, α4-, β1-, and β3-subunits and, therefore, the potential for α4β1 and αvβ3 integrins to frame the window of implantation, that that abnormal expression of the αv- and β3-subunits was correlated with human infertility. Failure of progesterone to downregulate the progesterone receptor in uterine epithelia was correlated with aberrant αv- and β3-subunit expression and infertility [129–132]. During this same period there were further reports on the expression of integrins in the human uterus that expanded the list of uterine integrin subunits to αv, α1, α2, α3, α4, α6, β1, β3, and β5 [52–57].

Demonstrations of integrin functions in human implantation cannot be performed in women and are limited to in vitro experiments. The Ishikawa human endometrial adenocarcinoma cell line [133] has been used extensively with human and/or rodent blastocysts or ligand-coated beads to examine ligand-integrin interactions relevant to implantation in humans. Expression of the αvβ3 integrin increases at sites of attachment of both mouse and human blastocysts to Ishikawa cells, and when the αvβ3 integrin, the αv-subunit, or the β3-subunit are knocked down in mouse blastocysts via siRNA, the stability of attachment of the blastocysts to Ishikawa cells decreases. Further, the attachment of OPN-coated microbeads to Ishikawa cells is inhibited when the αvβ3 integrin is knocked down in the Ishikawa cells [134]. Similarly, rat blastocysts do not attach to Ishikawa cells lacking the β3-subunit, and pre-incubation of either rat blastocysts or Ishikawa cells with RGD-blocking peptides significantly reduces attachment of the blastocysts to Ishikawa cells [135, 136].

The expression of integrins at the uterine-placental interface of humans is not limited to the peri-implantation period. The integrin subunits α3, αv, β1, β3, β4, and β5 are present on first trimester villous placenta with the α3-subunit localized to endothelia, the αv-subunit localized to the cytotrophoblasts, the β1-subunit localized to villous stromal cells, the β3-subunit localized to the apical surface of the syncytiotrophoblasts, the β4-subunit localized to the cytotrophoblasts, and the β5-subunit present on both cytotrophoblasts and syncytiotrophoblasts [57]. Anchored villous cytotrophoblasts of first trimester floating villi and placental bed biopsies immunostain for α6- and β4-subunits, the column cytotrophoblasts express α5- and β1-subunits, and α1-, α5- and β1-subunits are present on cytotrophoblasts clustered within the uterine wall. Indeed, the regulation of integrin expression may contribute to cytotrophoblast invasion into the decidua. Cytotrophoblast stem cells express the α6-subunit that is replaced by the α1-, α5- and β1-subunits in differentiating and invasive cytotrophoblasts [137]. When function-blocking antibodies directed against the α1- and α5-subunits were added to cytotrophoblast cells cultured in Matrigel, perturbation of the α1-subunit inhibited cytotrophoblast invasion whereas perturbation of the α5-subunit increased invasion [138].

Finally, IACs assemble in the myometrium of pregnant women to aid in the formation of a mechanical syncytium required for effective labor. Myometrial expression of integrin subunits αv, α5, α7, and β3 increases during gestation and α3-, α5-, α7- and β1-subunits colocalize with IAC proteins in the myometrium at term, suggesting that the α3β1, α5β1, and α7β1 integrins transmit mechanical signals from the ECM through IACs in the pregnant human myometrium [124]. Similar IACs have been reported in the myometrium of pregnant rats and sheep [122, 123, 125].

## Summary of integrin expression and function during gestation in rodents

The hatched mouse blastocyst lodges into a crypt on the antimesometrial side of the uterine lumen and the lumen closes around the blastocyst to form an implantation chamber [49]. The tight space formed by the implantation chamber restricts blastocyst movement and facilitates close apposition of the apical surfaces of trophoblast cells to uterine LE. The integrity of the implantation chamber is maintained via closure of the lumen surrounding the chamber. The mechanisms involved in closure of the uterine lumen at interimplantation sites in mice are not completely understood, but likely involve absorption of fluid within the uterine lumen mediated by uterine aquaporins [139–141]. Uterine LE apposed to the trophoblast undergo cell death or entosis soon after blastocyst attachment and primary trophoblast giant cells interface directly with the uterine stroma. Uterine LE adjacent to but not attached to the blastocyst degenerate a few days later leaving the conceptus embedded in the uterine wall [142]. Apposition of the mouse blastocyst occurs by day 4 of gestation when the blastocyst undergoes activation in response to a pulse of estrogen in order to initiate implantation on day 5 [143]. In mice, MUC1 is downregulated globally across the apical surface of the uterine LE in response to progesterone [144], and growth factors and other molecules secreted into the oviductal and uterine lumen stimulate trafficking of receptors to the trophoblast surface that are responsive to paracrine signals leading to juxtacrine interactions between the trophoblast and uterine LE culminating in integrin-mediated firm adhesion between these surfaces as the blastocysts migrate and encounter the basement membrane prior to invasion of the decidua [145].

Multiple integrin subunits are expressed by the mouse blastocyst. These include the constitutive expression of the αv-, α5-, α6-, β1- and β3-subunits and expression of the α2-, α6A- and α7- subunits in late blastocysts [55, 57]. The trafficking of integrins to the trophoblast surface is regulated by paracrine factors released into the uterine lumen including estrogen required for blastocyst activation, and this trafficking is involved in blastocysts becoming attachment competent. When mouse blastocysts are cultured with human Ishikawa cells, the blastocysts attach to the Ishikawa cells and upregulate the αvβ3 integrin at sites of attachment. When the αvβ3 integrin, the αv-subunit, or the β3-subunit are knocked down in mouse blastocysts via siRNA, blastocyst attachment to Ishikawa cells is less stable [134]. Further, preincubation of rat blastocysts or Ishikawa cells with RGD-blocking peptides reduces adhesion of blastocysts to Ishikawa cells, and rat blastocysts cultured with Ishikawa cells lacking the β3-subunit do not attach to the Ishikawa cells in vitro [135, 136]. Indeed, when mouse blastocysts are cultured with Ishikawa cells, the β3-subunit undergoes calcium-dependent translocation from the cytoplasm to the apical membrane of the trophoblast cells, and the trophoblast initiates RGD-dependent adhesion to the Ishikawa cells [146]. An elegant series of experiments reported by the Armant laboratory details integrin trafficking. In mouse blastocysts, the αvβ3 integrin is present at the apical surface of trophoblast cells. Binding of HB-EGF to receptors on the apical surface of trophoblast cells initiates calcium signaling that leads to trafficking of the α5β1 integrin to the apical surface. Ligation of FN1 to αvβ3 and α5β1 then promotes trafficking of the αIIbβ3 integrin to the apical surface to strengthen FN1 binding to trophoblast cells [146–149]. Integrin trafficking to the apical surface of uterine LE is also a feature of implantation in rodents. In rats the β1- and β3-subunits and TLN1 localize in IACs at the basal surface of cells on d 1 of gestation, but under the influence of progesterone these IACs disassemble, and the β3-subunit increases its expression at the apical surface of the uterine LE. Together these events are postulated to facilitate blastocyst attachment to the uterine LE and removal of the LE to facilitate blastocyst invasion to the decidua [150]. The importance of integrins to pregnancy in mice is highlighted by the fact that null mutations of the αv-, α5-, β1- or β5-subunits result in peri-implantation lethality and failure of chorioallantoic membrane fusion [151]. Further, functional blockade of αv- and β3-subunits in mice reduces the number of implantation sites [152]. Similarly, blocking of these subunits decreased the number of implantation sites in rabbits [153].

Finally, studies in rats have shown that expression of the α5-subunit mRNA and protein increases within the myometrium and the α5-subunit incorporates into IACs that assemble between adjacent smooth muscle cells during late pregnancy and labor [123]. Both hormones of pregnancy and mechanical stretch upregulate expression of FN1, the α5β1 integrin, and other IAC constituents, including the cytoskeletal mechanosensor TLN1 [119, 123, 153]. The growth of myometrial IACs is sensitive to rigidity and strength of adhesion to the ECM [154, 155] and the IAC linkage to the ECM and the myometrial actomyosin complex provides sufficient force to expel the fetus at term [122]. Similar IACs are detectable in the myometrium of pregnant humans and sheep [110, 124, 125].

## Conclusions

Integrins undoubtedly play significant roles during gestation in eutherian mammals. The complex nature of integrin structure, ligand binding, and inside-out and outside-in signaling, and their important roles as regulators of communication between cells and between cells and the ECM. These interactions allow integrins to be key mediators in many of the tissue remodeling events that occur during early embryonic development, implantation, formation of the placenta, and myometrial contractility. Although domestic animal models for research are often underappreciated [156], the vast variations among the placentae of different species necessitate significant variations in the expression and function of integrins at the uterine-placental interface, and this highlights the value of comparative studies in the field of integrins and placentation. Our understanding of the complex physiology of integrins and their roles during pregnancy has been, and will likely continue to be, advanced by studies of pigs, sheep, humans and rodents as animal models for biomedical research. This field of research is an excellent example of how the “boat” of our understanding rises as laboratories focusing on different species take note of the advances made by one-another and build on those legacies.

## Data Availability

Not applicable.

## Abbreviations

CL: Corpus luteum
ECM: Extracellular matrix
EGF: Epidermal growth factor
EPC: Endothelial progenitor cells
ERK: Extracellular signal regulated kinase
FAK: Focal adhesion kinase
FN1: Fibronectin
FRAP1: FK506-binding protein 12-rapamycin-associated protein1
GE: Glandular Epithelium
GFOGER: Glycine-phenylalanine-hydroxyproline-glycine-glutamic acid-arginine
GlyCAM-1: Glycosylation dependent cell adhesion molecule 1
HB-EGF: Heparin-binding epidermal-like growth factor
HUVEC: Human umbilical vein endothelial cell
IAC: Integrin adhesion complex
IαIH4: Inter-α-trypsin inhibitor heavy chain-like protein
ICM: Inner cell Mass
ILK: Integrin-linked kinase
IFNT: Interferon tau
LAP: Latency associated peptide
LDV: Leucine-aspartic acid-valine
LE: Luminal epithelium
LGALS15: Galectin 15
MAPK: Mitogen-activated protein kinase
MIDAS: Metal ion-dependent adhesion site
MTOR: Mechanistic target of rapamycin
MUC1: Mucin 1
NOS3: Nitric oxide synthase 3
OPN: Osteopontin
oTr1: Ovine trophoblast 1 cells
P70S6K: P70 ribosomal protein S6 kinase beta-1
PINCH: Particularly interesting new cysteine-histadine-rich protein
pTr2: Porcine trophoblast 2 cells
pUE: Porcine uterine epithelial cells
RGD: Artinine-glycine-aspartic acid
sGE: Shallow glandular epithelium
SPP1: Secreted phosphoprotein 1
TGFβ: Transforming growth factor beta
TLN1: Talin
VASP: Vasodilator-stimulated phosphoprotein
VCL: Vinculin
VTN: Vitronectin
